# Anti-Inflammatory Effects of Resveratrol, Curcumin and Simvastatin in Acute Small Intestinal Inflammation

**DOI:** 10.1371/journal.pone.0015099

**Published:** 2010-12-03

**Authors:** Stefan Bereswill, Melba Muñoz, André Fischer, Rita Plickert, Lea-Maxie Haag, Bettina Otto, Anja A. Kühl, Christoph Loddenkemper, Ulf B. Göbel, Markus M. Heimesaat

**Affiliations:** 1 Institut für Mikrobiologie und Hygiene, Charité – Universitätsmedizin Berlin, Berlin, Germany; 2 Institut für Pathologie, Charité - Universitätsmedizin Berlin, Berlin, Germany; University of Hyderabad, India

## Abstract

**Background:**

The health beneficial effects of Resveratrol, Curcumin and Simvastatin have been demonstrated in various experimental models of inflammation. We investigated the potential anti-inflammatory and immunomodulatory mechanisms of the above mentioned compounds in a murine model of hyper-acute Th1-type ileitis following peroral infection with *Toxoplasma gondii*.

**Methodology/Principal Findings:**

Here we show that after peroral administration of Resveratrol, Curcumin or Simvastatin, mice were protected from ileitis development and survived the acute phase of inflammation whereas all Placebo treated controls died. In particular, Resveratrol treatment resulted in longer-term survival. Resveratrol, Curcumin or Simvastatin treated animals displayed significantly increased numbers of regulatory T cells and augmented intestinal epithelial cell proliferation/regeneration in the ileum mucosa compared to placebo control animals. In contrast, mucosal T lymphocyte and neutrophilic granulocyte numbers in treated mice were reduced. In addition, levels of the anti-inflammatory cytokine IL-10 in ileum, mesenteric lymph nodes and spleen were increased whereas pro-inflammatory cytokine expression (IL-23p19, IFN-γ, TNF-α, IL-6, MCP-1) was found to be significantly lower in the ileum of treated animals as compared to Placebo controls. Furthermore, treated animals displayed not only fewer pro-inflammatory enterobacteria and enterococci but also higher anti-inflammatory lactobacilli and bifidobacteria loads. Most importantly, treatment with all three compounds preserved intestinal barrier functions as indicated by reduced bacterial translocation rates into spleen, liver, kidney and blood.

**Conclusion/Significance:**

Oral treatment with Resveratrol, Curcumin or Simvastatin ameliorates acute small intestinal inflammation by down-regulating Th1-type immune responses and prevents bacterial translocation by maintaining gut barrier function. These findings provide novel and potential prophylaxis and treatment options of patients with inflammatory bowel diseases.

## Introduction

Several nutritional compounds are the focus of public attention because of their potential beneficial health effects. Some compounds derived from plants and herbs (e.g. Resveratrol, Curcumin) have been proven effective in traditional medicine as herbal remedies for inflammatory disorders such as hepatitis, arthritis, and colitis [1]. Both, Resveratrol and Curcumin also exert pharmacological effects in various experimental models of acute and chronic inflammation [1]. In addition, some pharmaceutical compounds (e.g. statins) have shown to possess anti-inflammatory, immuno-modulatory properties apart from their original indication for disease treatment [2].

Resveratrol (3,5,4′-trihydoxy-*trans*-stilbene) is a polyphenol found in grape skins and red wine. Resveratrol has been reported to exert multiple health-promoting benefits such as anti-inflammatory, anti-oxidant, anti-tumor, anti-platelet aggregation, anti-aging and anti-atherogenic effects [3]. Anti-inflammatory properties of Resveratrol have been described in several diseases such as arthritis [4], pancreatitis [5], and experimental colitis [6].

Curcumin is a polyphenol derived from the root of the turmeric plant *Curcuma longa* and responsible for the yellow-orange color and the spicy taste of curries [7]. Curcumin exerts anti-infectious, anti-tumor, and anti-inflammatory properties [8]. Preventive as well as therapeutic anti-inflammatory effects of Curcumin treatment have been observed in various animal models [9].

Simvastatin belongs to the class of cholesterol-lowering statins that are widely used to reduce cardiovascular morbidity and mortality in patients with or without coronary artery disease [10]. Statins have been also proposed to exert anti-inflammatory effects that are not directly related to their cholesterol-lowering activity [2] by interfering with endothelial adhesion and leukocyte migration to sites of inflammation [11].

Within eight to ten days following peroral infection with the parasite *Toxoplasma (T.) gondii*, susceptible mice develop severe small intestinal necrosis (pan-ileitis), induced by IL-23 [12] and mediated by a strong Th1-type immune response with a subsequent storm of pro-inflammatory cytokines including IL-12, IFN-γ, TNF-α, and nitric oxide (NO) [13]. Furthermore, inflammation triggers characteristic quantitative as well as qualitative ileum flora shifts [14], [15]. We demonstrated earlier that luminal concentrations of commensal Gram-negative bacteria such as *E. coli* increase and aggravate ileitis development through TLR4-dependent signaling pathways [15]. Moreover, commensal gut bacteria translocate through the leaky epithelial barrier into the intestinal submucosa and spread to adjacent organs through blood or lymphatic vessels leading to sepsis and multi-organ failure [12]. Taken together, the Th1-type immunopathology in the *T. gondii*-mediated ileitis model is triggered by the commensal microbiota and thus resembles the immunological key features of acute episodes in inflammatory bowel disease (IBD) such as Crohn's disease (ileitis terminalis) in humans. However, experimental models of small intestinal inflammation are scarce and most of the experimental IBD models assess the large intestine. Given that Resveratrol, Curcumin or Simvastatin have been shown to suppress immune responses, we investigated the immuno-modulatory effects of these compounds in this well characterized model of ileitis. Our results showed the anti-inflammatory and immuno-modulating properties of all three compounds. Amelioration of ileitis was accompanied by reduced immunopathology and an integral gut barrier function that prevented bacterial translocation. These findings provide novel potential therapeutic options for patients during the acute episodes of Crohn's disease.

## Results

### Resveratrol treatment prolongs survival in an acute ileitis model

**Figure 1:**
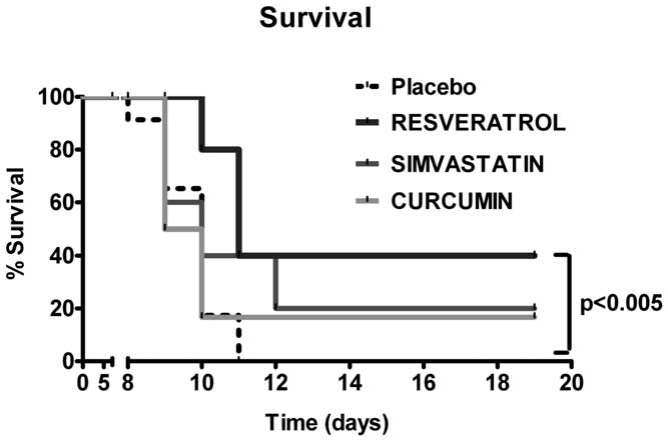
Survival rates of mice treated with Placebo (n = 23), Simvastain (n = 5), Resveratrol (n = 5) or Curcumin (n = 6) from d-2 until d8 p.i.. Mortality of animals was monitored daily until d19 p.i.. Significance levels (as compared with Placebo controls) were determined by Kaplan-Meier analysis.

To investigate the beneficial effects of Resveratrol, Curcumin, and Simvastatin on small intestinal inflammation, we infected C57BL/10 mice with *T. gondii* and compared survival rates between treated and Placebo control mice. Whereas all untreated control mice had died at day 11 p.i., 20% of Curcumin- and 40% of Simvastatin- or Resveratrol-treated animals survived the acute phase of inflammation (**Fig. 1**). Furthermore, Resveratrol treated mice displayed significantly higher survival rates until the end of the experiment as compared to Placebo controls (40% survival, 19 days post infection; p<0.005, Kaplan Meier analysis; **Fig. 1**). Thus, Resveratrol treatment prolongs survival in our hyper-acute ileitis model.

### Simvastatin, Curcumin, and Resveratrol ameliorate acute small intestinal inflammation

**Figure 2:**
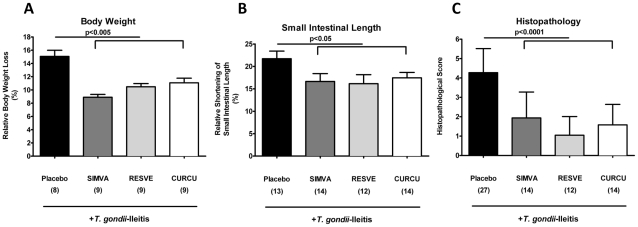
Less small intestinal immunopathology after treatment with Simvastain, Resveratrol or Curcumin (8 days after *T. gondii* infection). (**A**) Relative body weight loss of mice after treatment with Placebo (n = 8), Simvastain (SIMVA; n = 9), Resveratrol (RESVE; n = 9) or Curcumin (CURCU; n = 9) from d-2 until d8 p.i. (**B**) Relative small intestinal shortening of mice after treatment with Placebo (n = 13), Simvastain (SIMVA; n = 14), Resveratrol (RESVE; n = 12) or Curcumin (CURCU; n = 14) from d-2 until d8 p.i. (**C**) Histopathology of the terminal ileum of mice treated with Placebo (n = 27), Simvastain (SIMVA; n = 14), Resveratrol (RESVE; n = 12) or Curcumin (CURCU; n = 14) from d-2 until d8 p.i.. Mean values, standard deviations, and significance levels as indicated were determined by the Student's t test. Data are pooled from at least three independent experiments.

Mice orally treated with Simvastatin, Resveratrol or Curcumin displayed significantly less macroscopic signs of intestinal inflammation. Within 8 days p.i., Placebo control mice had lost approximately 15% of their initial body weight whereas mice treated with either compound displayed significant less weight loss (between 9 and 11%; p<0.005) (**Fig. 2 A**). Given that ileal inflammation is accompanied by a significant shortening of the upper intestinal tract, we determined the lengths of the small intestine between treated and control animals. *T. gondii* infection resulted in less significant shortening of the small intestinal length in treated mice compared with the Placebo group (16–18% vs. 22% respectively; p<0.05) (**Fig. 2 B**). Moreover, animals treated with Simvastatin, Resveratrol or Curcumin displayed only mild signs of inflammation in their ileal mucosa including edema and cell-free exudate into the lumen but maintained an intact epithelium. In contrast, severe ileal inflammation including initial necrosis was observed in the control group (p<0.0001) (**Fig. 2 C**). Thus, oral treatment of Simvastatin, Resveratrol or Curcumin ameliorates acute small intestinal *T. gondii*-induced inflammation.

To examine whether differences in *T. gondii* loads within the ileum might have contributed to the beneficial effects observed after peroral treatment with Simvastatin, Resveratrol or Curcumin, we assessed *T. gondii* DNA concentrations in treated and untreated mice. Neither the amount of *T. gondii* DNA nor the number of parasitophorous vacuoles containing *T. gondii* tachyzoites in the ileum differed significantly between treated and Placebo groups at the same time point (data not shown).

### Immuno-modulatory effects of Simvastatin, Resveratrol or Curcumin treatment in the mucosa *in situ* during acute ileitis

**Figure 3:**
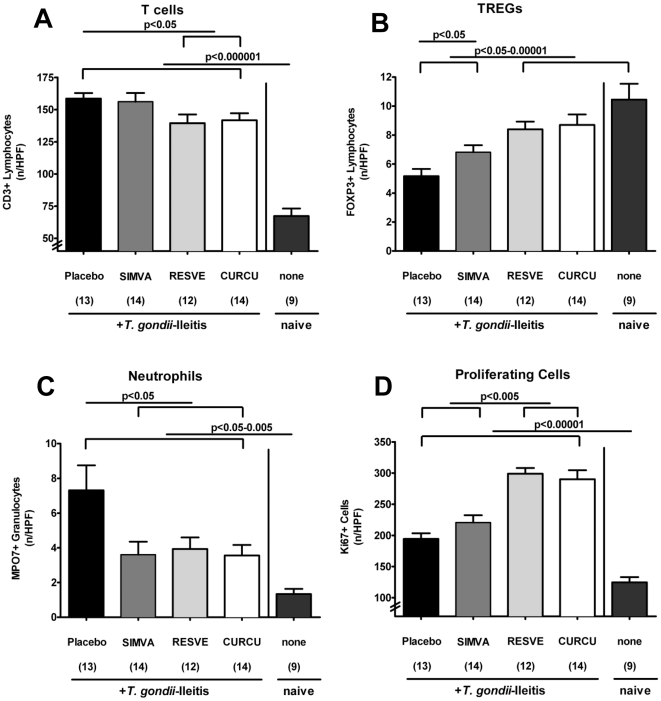
Quantification of defined cell population in the ileum of mice *in situ* after treatment with Simvastain, Resveratrol or Curcumin (8 days after *T. gondii* infection). The average number of cells positive for (**A**) CD3, (**B**) FOXP3, (**C**) MPO7 (Myeloperoxidase), and (**D**) Ki-67 from at least six high power fields (HPF, 400× magnification) per animal were determined microscopically in immunostained ileum sections at d8 p.i. after treatment of mice with Placebo, Simvastain (SIMVA), Resveratrol (RESVE), or Curcumin (CURCU) from d-2 until d8 p.i. and naïve, uninfected animals. Numbers of analyzed animals are given in parentheses. Mean values, standard errors of the mean, and significance levels as indicated were determined by the Student's t test. Data are pooled from at least three independent experiments.

To further elucidate potential immuno-modulatory mechanisms by which the compounds under investigation exerted their beneficial effects, we assessed the number of several immune cell subsets in the ileal mucosa and submucosa of treated vs control mice with ileitis (**Fig. 3 A–D**). Because acute inflammation in *T. gondii* ileitis is T-cell driven and Th1 dominated [13], and there is strong evidence that regulatory T cells accumulate at inflamed tissue sites in IBD patients [16], we quantified CD3^+^ and FOXP3^+^ T cells in the ileum *in situ* by immunohistochemistry. Ileitis resulted in a significant accumulation of CD3^+^ cells within the ileum mucosa and submucosa (p<0.000001 vs naïve mice) (**Fig. 3 A**). This increase of T lymphocytes, however, was significantly less pronounced in mice treated either with Resveratrol or Curcumin (p<0.05 vs. Placebo) (**Fig. 3 A**). Furthermore acute ileitis was accompanied by a 50% reduction of FOXP3^+^ T lymphocytes in the ileum on d8 p.i. (p<0.00001 as compared to non-infected, naïve animals, **Fig. 3 B**). Most importantly, mice from either the Simvastain, Resveratrol or Curcumin treatment group displayed 20–30% higher FOXP3^+^ cell numbers as compared to Placebo controls with ileitis on d8 p.i. (p<0.05-0.00001) (**Fig. 3 B**).

Given that neutrophilic granulocytes are important effector innate immune cells during acute ileitis development, we investigated the recruitment of myeloperoxidase-7^+^ (MPO-7^+^) cells in the small intestinal lamina propria of both treated and control mice in the acute phase of disease (**Fig. 3 C**). On d8 p.i., a more than 5-fold increase in MPO-7^+^ cells was observed within the ileum of Placebo control animals compared to naïve mice, which were non-infected and untreated (p<0.005). However, Simvastatin, Resveratrol or Curcumin treated mice displayed 25–50% fewer MPO-7^+^ cells as compared to Placebo controls (p<0.05) (**Fig. 3 C**).

In addition, we evaluated cell proliferation and regeneration in ileal sections on d8 p.i. using Ki-67, an antibody that recognizes a nuclear cell proliferation-associated antigen expressed in all active stages of the cell cycle [17]. After *T. gondii* infection, Ki-67^+^ cell numbers increased significantly as compared to naïve mice (p<0.00001) (**Fig. 3 D**). Mice treated with either Resveratrol or Curcumin, however, displayed 50% more Ki-67^+^ cells than Simvastatin- or Placebo treated animals on d8 p.i. (p<0.005).

Taken together, treatment with Simvastatin, Resveratrol or Curcumin modulates small intestinal immune cell responses during acute ileitis by decreasing CD3^+^ and MPO7^+^ cell accumulation but increasing FOXP3^+^ and Ki-67^+^ cell numbers.

### Pro- and anti-inflammatory cytokine responses after peroral treatment of mice with acute ileitis

Given that treated mice were resistant to the development of *T. gondii*-induced ileitis compared to untreated mice but *T. gondii* DNA concentration in the ileum did not differ significantly between these groups, we hypothesized that differential pro-inflammatory cytokine expression profiles within the ilea, mesenteric lymph nodes (MLNs) or spleens might explain the reduced immunopathology observed after treatment with the compounds mentioned above at d8 p.i..

**Figure 4:**
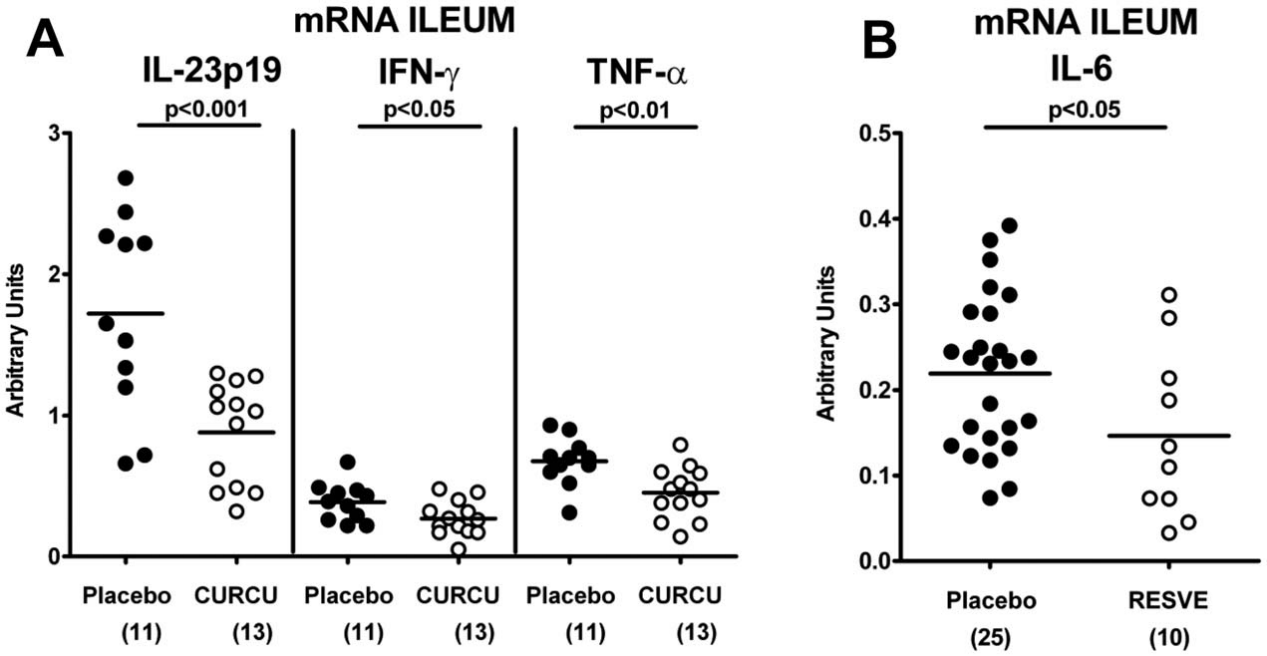
m-RNA Expression of pro-inflammatory cytokines in ilea of mice 8 days after *T. gondii* infection and treatment with (A) Curcumin or (B) Resveratrol from d-2 until d8 p.i. compared to Placebo controls. RT-PCR results of IL-23p19, IFN-γ and TNF-α (**A**) and IL-6 (**B**) expression from individual mice are expressed as fold changes relative to HPRT mRNA expression (arbitrary units). Numbers of analyzed animals are given in parentheses. Mean values and significance levels as indicated were determined by the Student's t test. Data are pooled from at least three independent experiments.

**Figure 5:**
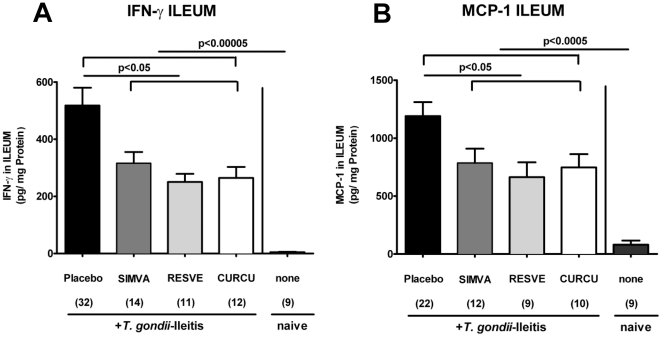
(A) IFN-γ and (B) MCP-1 concentrations (CBA-ELISA) in supernatants of ileum cultures from naïve, uninfected animals and mice treated with Simvastatin (SIMVA), Resveratrol (RESVE) or Curcumin (CURCU) from d-2 until d8 p.i. as compared to Placebo controls (8 days after *T. gondii* infection). Numbers of analyzed animals are given in parentheses. Mean values, standard errors of the mean, and significance levels as indicated were determined by the Student's t test. Data are pooled from at least two independent experiments.

**Figure 6:**
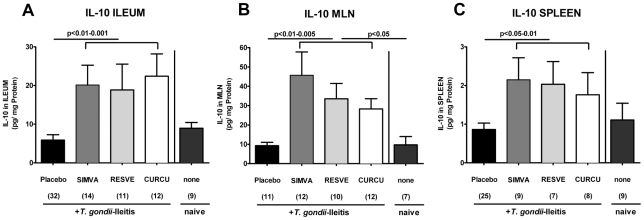
IL-10 concentrations (CBA-ELISA) in supernatants of Ileum (A), MLN (B) and spleen (C) cultures from naïve, uninfected animals and mice treated with Simvastatin (SIMVA), Resveratrol (RESVE) or Curcumin (CURCU) from d-2 until d8 p.i. as compared to Placebo controls (8 days after *T. gondii* infection). Numbers of analyzed animals are given in parentheses. Mean values, standard errors of the mean, and significance levels as indicated were determined by the Student's t test. Data are pooled from at least two independent experiments.

The analysis of mRNA in the ileum *in situ* revealed that IL-23p19 (p<0.001), IFN-γ (p<0.05), and TNF-α (p<0.01) expressions were significantly down-regulated in Curcumin-treated mice at d8 p.i. as compared to untreated controls (**Fig. 4 A**), whereas Resveratrol treated animals exhibited lower IL-6 ileal mRNA expression (**Fig. 4 B**). In addition, IFN-γ (p<0.05) and MCP-1 (p<0.05) secretion into *ex vivo* ileum cultures were significantly lower after Resveratrol, Curcumin or Simvastatin treatment compared to Placebo (**Fig. 5 A–B**). Furthermore, significantly higher amounts of the anti-inflammatory cytokine IL-10 were found in the ilea (p<0.01-0.001), MLNs (p<0.01-0.005) and spleens (p<0.05-0.01) after either treatment regimen compared to Placebo controls with ileitis (**Fig. 6 A–C**).

Taken together, our results show that treatment with either Simvastain, Resveratrol or Curcumin modulates the cytokine profile within the small intestine which agrees with changes of immune cell subsets (fewer T cells and neutrophils, more Tregs) observed in treated mice pointing towards less pro- and more anti-inflammatory responses.

### Reduced pro-inflammatory gut bacterial species in the small intestine of mice treated with Simvastatin, Curcumin or Resveratrol

**Figure 7:**
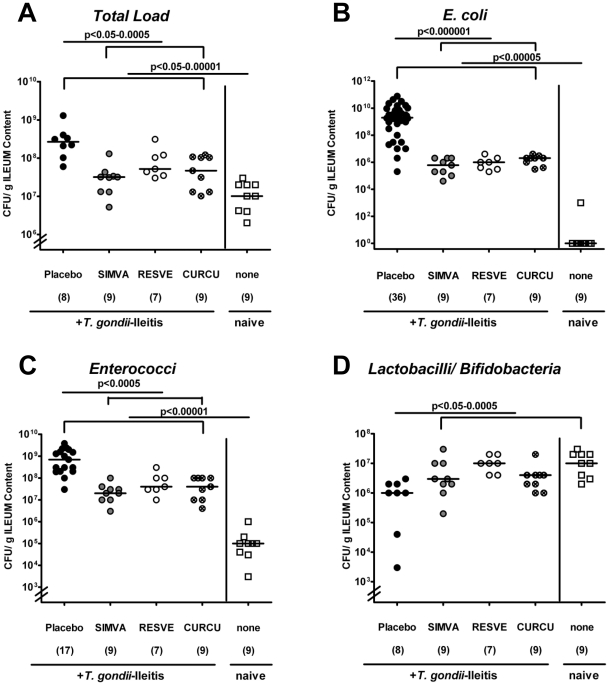
Changes in the composition of the luminal ileum flora obtained from uninfected animals (none) and *T. gondii*-infected mice treated with Simvastain (SIMVA), Resveratrol (RESVE) or Curcumin (CURCU) from d-2 until d8 p.i. as compared to Placebo controls (8 days after *T. gondii* infection). Detailed cultural analysis was performed on appropriate selective media, bacterial species identified by biochemical analysis and reconfirmed by comparative sequence analysis (see Methods). Individual bacterial counts (CFU, colony forming units) of (**A**) Total bacterial load, (**B**) *E. coli*, (**C**) enterococci, (**D**) lactobacilli/bifidobacteria obtained from respectively treated mice (numbers of animals are given in parentheses), medians and levels of significance determined by Mann-Whitney-U test are indicated. Data are pooled from two representative experiments.

Because we have shown that *E. coli* overgrowth in the terminal ileum aggravates *T. gondii* induced inflammation [14], [15], we compared the composition of the ileum microflora of mice after treatment with Simvastatin, Curcumin or Resveratrol at d8 p.i.. Cultural analysis demonstrated that the total bacterial load increased during ileitis, but was significantly reduced by 1 to 2 orders of magnitude in treated mice as compared to Placebo controls (**Fig. 7 A**) (p<0.05-0.0005). A more detailed cultural analysis revealed that following treatment concentrations of pro-inflammatory bacterial species such as *E. coli* (**Fig. 7 B**) and enterococci (**Fig. 7 C**) were significantly reduced by 3.0 – 3.5 (p<0.000001) and 1.0 – 1.5 (p<0.0005) orders of magnitude, respectively, whereas the potentially anti-inflammatory lactobacilli and bifidobacteria populations were slightly increased by up to 1.0 log orders (**Fig. 7 D**) (p<0.05-0.0005). Counts of *Clostridium* and *Eubacterium* spp. were unaffected after treatment (data not shown). Interestingly, changes in the ileal flora composition were comparable in the respective treatment groups. Furthermore, a direct antimicrobial effect of Simvastatin, Resveratrol or Curcumin was ruled out by specific *in vitro* testing of the substances in the presence of the highly susceptible laboratory strain *Bacillus subtilis* (data not shown). Thus, shifts in bacterial ileal flora concentrations (lower bacterial loads and less *E. coli* overgrowth) in treated mice during ileitis were not due to direct antimicrobial effects but rather agrees with the observed macroscopic and microscopic anti-inflammatory effects of the respective substances.

### Simvastatin, Curcumin or Resveratrol treatment prevents bacterial translocation during *T. gondii*-induced ileitis

**Figure 8:**
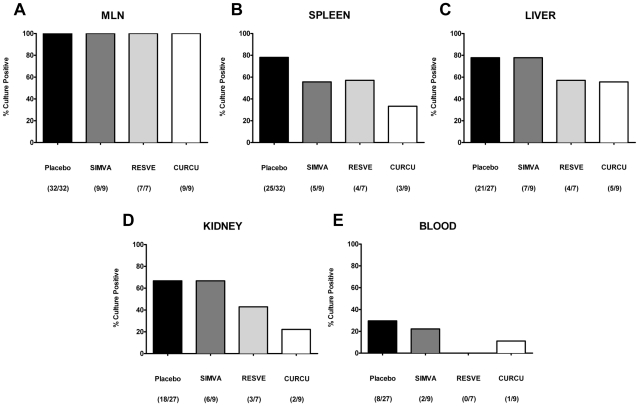
Bacterial translocation into (A) MLN, (B) Spleen, (C) Liver, (D) Kidney, and (E) Blood of mice treated with Simvastain (SIMVA), Resveratrol (RESVE) or Curcumin (CURCU) from d-2 until d8 p.i. as compared to Placebo controls (8 days after *T. gondii* infection). Organs or blood were cultivated in thioglycolate broth for maximum 7 days. Turbid broths were sub-cultivated on solid media (refer to Materials). Indicated are relative rates of positive samples (in %) and absolute numbers of positive samples out of total number analyzed in parentheses.

Since we have recently demonstrated that acute ileitis is accompanied by translocation of living bacteria from the commensal gut flora into adjacent organs [15] we lastly investigated translocation rates following Resveratrol, Simvastatin, or Curcumin treatment. Bacterial translocation into MLNs was detected in all animals at day 8 p.i.. (**Fig. 8 A**). Living bacteria translocated into the spleen in 78.1%, 57.1%, 55.6%, or 33.3% of cases after treatment with Placebo, Resveratrol, Simvastatin, or Curcumin, respectively (**Fig. 8 B**). The translocation rates into liver (**Fig. 8 C**) and kidney (**Fig. 8 D**) were much lower in Resveratrol- (57.1% and 42.9%, respectively) and Curcumin- (55.6% and 22.2%, respectively) treated animals as compared to the Placebo (77.8% and 66.7%, respectively) and Simvastatin (77.8% and 66.7%, respectively) groups. Oral treatment with Simvastatin (22.2%) or Curcumin (11.1%) resulted in less frequent bacteremia (and thus subsequently less frequent potential lethal sepsis) as compared to Placebo controls (29.6%) at d8 p.i. (**Fig. 8 E**). Surprisingly, all blood samples taken from Resveratrol treated mice bacteria remained sterile. The translocating bacterial species that could be cultured from the organs were predominantly *E. coli* and enterococci, the bacterial groups that were overgrowing the ileum lumen during ileitis as described above. No living bacteria could be cultured from the respective organs of naïve (non-infected, untreated) mice with intact small intestinal barrier function.

Thus, Simvastatin, Curcumin and Resveratrol treatment remarkably reduced bacterial translocation into subepithelial tissues thereby minimizing bacteria/TLR ligation and preventing immune cell activation and subsequent inflammatory response initiation.

## Discussion

Inflammatory bowel diseases (IBD) such as Crohn's disease (also termed ileitis terminalis) and ulcerative colitis are characterized by chronic intestinal inflammation with acute episodes of multi-factorial etiology [18], [19]. The vast majority of experimental studies concerning intestinal inflammation have been performed in murine models affecting the colon. Thus, we used our well-established, hyper-acute model of *small* intestinal inflammation following *T. gondii* infection to investigate anti-inflammatory and immuno-modulatory effects of Resveratrol, Curcumin, and Simvastatin in ileal inflammation. The immunopathology observed after *T. gondii* infection is driven by Th1 cells and by the bacterial gut flora. This situation resembles immunopathology during acute episodes of Crohn's disease [20]. Furthermore, the underlying immunopathology is characterized by an overproduction of pro-inflammatory cytokines such as IL-12, IFN-γ, TNF-α, and IL-23, a key regulator of the immune response cascade [12], [13], [21].

Here, we demonstrate that hyper-acute inflammation in the *small* intestine is ameliorated by either Resveratrol, Curcumin or Simvastatin treatment. In addition, the anti-inflammatory effects of the compounds under investigation during the acute phase of ileitis (on day 8 after ileitis induction by peroral infection with *T. gondii*) were comparable: animals from all three treatment groups were protected from immunopathology development and exhibited better clinical conditions, less body weight loss and shortening of the small intestinal length. Untreated mice also displayed severe pan-ileitis whereas treated animals (irrespective of the compound used) only showed minor mucosal changes with an intact epithelial lining. Furthermore, reduced ileal inflammation in Resveratrol and Curcumin treated mice might result from compensatory higher proliferation rates of intestinal epithelial cells found in the ileal mucosa as shown by higher Ki67^+^ cell numbers after respective treatment.

Moreover, the amelioration of immunopathology was accompanied by a reduced accumulation of CD3^+^ T lymphocytes as well as lower expression of the key regulator IL-23 and pro-inflammatory cytokines IFN-γ and TNF-α in the small intestinal lamina propria after Resveratrol or Curcumin treatment. Furthermore, ileal secretion of IFN-γ and the monocyte chemotactic protein MCP-1 was significantly decreased after treatment with Simvastatin, Resveratrol, or Curcumin. In parallel, myeloperoxidase-7^+^ neutrophilic granulocytes recruitment was diminished thereby limiting oxidative stress in the ileum and maintaining an integral epithelial cell barrier function. In addition, numbers of FOXP3^+^ cells were diminished in untreated mice, but significantly increased (comparable to the observed numbers in naïve mice) in treated animals after ileitis induction which correlates with increased concentrations of the anti-inflammatory cytokine IL-10 observed in ileum, MLNs, and spleen at d8 p.i..

Simvastatin has been shown to inhibit acute as well as chronic inflammatory responses in a cholesterol-independent manner by interfering with endothelial adhesion and leukocyte migration to sites of inflammation [11], [22]. In rats with normal blood cholesterol levels, simvastatin was found to ameliorate immunopathology in an acute TNBS colitis model by blocking neutrophil accumulation in the small intestine and lowering serum TNF-α levels [23].

Curcumin exerts its anti-inflammatory effects in murine colitis models by inhibiting COX2- and pro-inflammatory cytokine expression such as nitric oxide [9], [24], [25] and suppressing NF-κB activation [26], [27].

In acute TNBS rat colitis model, a decreased neutrophil influx and IL-1β production as well as an enhanced apoptosis of colonic mucosa was observed following Resveratrol treatment [28]. In chronic DSS-induced colitis, both decreased expression of pro-inflammatory cytokines (TNF-α, NO, and IL-1β) and increased anti-inflammatory mediators (IL-10) in the colonic mucosa contributed to attenuate inflammation after Resveratrol treatment [29]. In addition, even low dose Resveratrol treatment exerted anti-inflammatory effects in an acute DSS-induced colitis model by reducing expression levels of prostaglandin-E2, COX2, and NO [6].

Changes in commensal gut flora composition observed in acute *T. gondii*-induced ileitis resemble the gut flora changes found in humans with IBD [14], [15]. Shifts in the ileal flora composition become overt around d5 p.i., the time point when first significant defects of the epithelial barrier are histologically detectable. Ileitis development is accompanied by decreased species diversity, decreased loads of potentially anti-inflammatory species such as lactobacilli and bifidobacteria and a marked increase in commensal, potentially pro-inflammatory *E. coli* in the ileum lumen by 6–8 orders of magnitude. Furthermore, overgrowing *E. coli* within the gut aggravates Toll-like receptor- (TLR-) 4 driven immune responses during *T. gondii*-induced ileitis [15]. In addition, overgrowing bacteria easily translocate through a disrupted epithelial cell barrier into the intestinal lamina propria, lymphoid tissues (e.g. the ileum draining MLNs) and subsequently come in contact with immune cells thereby exacerbating intestinal inflammation. Lastly, when translocating bacteria reach the systemic blood stream, peritonitis, sepsis and subsequent multiple organ failure occurs with fatal consequences [14], [15]. Therefore, we investigated whether Simvastatin, Resveratrol or Curcumin could prevent gut flora composition changes and bacterial translocation after *T. gondii* infection. Interestingly, treatment with either compound significantly diminished total bacterial ileal burdens as compared to untreated animals. Strikingly, lower *E. coli* and enterococci counts were found in treated mice whereas lactobacilli and bifidobacteria populations were slightly but significantly increased. In addition, ileitis development is accompanied by a marked decrease in the lactobacilli and bifidobacteria populations [14] and strikingly, after either treatment these lactobacilli and bifidobacteria populations remained at comparable levels with naïve, uninfected mice. Remarkably, none of the compounds had a direct anti-microbial effect *in vitro*.

Larrosa and co-workers reported in 2009 similar flora shifts in an acute DSS-induced colitis model, however, to a much lesser extent: After low-dose Resveratrol treatment, rats exhibited increases in lactobacilli and bifidobacteria populations within the colonic lumen and blocked the increase in enterobacteria (mainly *E. coli*) [6]. In several studies, a protective effect of lactobacilli and bifidobacteria in DSS-colitis could be demonstrated [30], as well as modulatory effects upon pro-inflammatory responses in intestinal epithelial cells that have been challenged with pathogenic enterobacteria [31]. Thus, abundance of anti-inflammatory lactobacilli and bifidobacteria together with scarcity of pro-inflammatory *E. coli* markedly limit inflammation during *T. gondii* ileitis.

Resveratrol, Simvastatin, or Curcumin treatment might have an anti-inflammatory effect by directly interfering with the TLR/MyD88 signaling pathway. Recent findings in acute TNBS-induced colitis model in rats demonstrate that Curcumin-induced inhibition of intestinal inflammation involves TLR-4 and MyD88 [32].

Furthermore, an effect of Curcumin on matrixmetalloproteinases (MMPs; important in the pathogenesis of human IBD [33]) was recently described [34]: Curcumin down-regulated MMP-9 (gelatinase-B) activity on inflammation-induced intestinal epithelial cells *in vitro*. We have recently shown that non-selective MMP- as well as selective gelatinase blockage by a synthetic compound ameliorated *T. gondii* ileitis [12]. However, MMP-2, but not MMP-9 played a crucial role in mediating small intestinal pathology. MMP-9 is proposed to play an important role in experimental colitis [35]. Interestingly, neutrophils have been proposed as an important source for MMPs. Thus, inhibition of neutrophil migration by the investigated compounds might also down-modulated MMP secretion thereby diminishing epithelial cell inflammation.

In a recent study, Curcumin has been shown to inhibit oxidative stress [36] that in turn has been associated with tight junction opening, thereby modifying intestinal permeability [37]. In the present study, bacterial translocation rates (due to compromised epithelial barrier function) into spleen and cardiac blood after treatment with either compound were lower as compared to the Placebo group. Therefore, Resveratrol, Simvastatin, and Curcumin might modulate tight junction protein expression and function.

Most strikingly, 20 to 40% of the animals treated with Resveratrol, Simvastatin, or Curcumin survived the acute phase of inflammation whereas all untreated mice had died at d11 p.i.. In addition, 40% of Resveratrol treated mice displayed a long-term survival (until the end of the observation period until d19 p.i.; p<0.005 vs. Placebo). The long-term survival effect of Resveratrol is remarkable for three reasons: First, in our study, the duration of treatment (before and during course of acute inflammation) was rather short: Resveratrol treatment was initiated two days prior to ileitis induction and lasted 10 days in total; at d8 p.i. treatment was withdrawn. Second, given that the bioavailibility of the compound is low, short-term and local rather than systemic effects are observed within the gut lumen (effective until d8/d9 p.i.). Because *T. gondii*-induced ileitis compromises the intestinal absorption capacity, Resveratrol absorption after oral application is also reduced. Third, Resveratrol treatment improves survival in a hyper-acute small intestinal inflammation model where all Placebo control mice died. Thus far, Resveratrol treatment has only been shown to reduce mortality in a chronic colitis model [29]. We might hypothesize that Resveratrol treatment prevents bacterial translocation into the systemic circulation because in our study, we did not observe bacterial growth in any of blood cultures drawn from Resveratrol treated mice. Therefore, mice are protected from a bacteremia-induced sepsis that leads to death. Furthermore, fewer bacteria translocation rates into sub-epithelial tissues might be due to a less compromised epithelial barrier function. Fewer translocation rates are mirrored by an IL-6 decrease in the ileum of Resveratrol treated animals. Our findings agree with those in the literature suggesting that genes belonging to the IL-6 pathway are down-regulated in Resveratrol treated mice in experimental colitis [6] and other inflammation models [38].

In conclusion, here we show for the first time that Resveratrol, Curcumin, and Simvastatin exert potent anti-inflammatory effects in a hyper-acute Th1-type driven small intestinal inflammation model. In addition, Resveratrol, Curcumin, and Simvastatin represent novel and promising intervention strategies for prophylaxis and therapy of small intestinal inflammation in IBD.

## Materials and Methods

### Ethics Statement

All animal experiments were conducted according to the German animal protection laws (LaGeSo Berlin, TVV G0145/10 and G0146/10) with approval from the Charité – Universitätsmedizin ethical committee. Animal welfare was monitored twice daily by assessment of clinical conditions and weight loss of mice.

### Mice, parasites and ileitis induction

C57BL/10ScSn (wildtype) mice used for experiments were 3 months old and bred under specific pathogen-free (SPF) conditions in the Forschungsinstitut für Experimentelle Medizin (Charité, Berlin, Germany). For induction of ileitis, mice were infected orally with 100 *T. gondii* cysts (ME49 strain) from homogenized brains of intraperitoneally infected NMRI mice in a volume of 0.3 ml phosphate-buffered saline (PBS, pH 7.4) by gavage, as described previously [14].

### Compounds

Resveratrol, Curcumin, and Simvastatin were dissolved in 2% Carboxy-Methyl-Cellulose (in PBS; all purchased from Sigma-Aldrich, München, Germany) and administered to mice with a daily dose of 20 mg, 100 mg, and 20 mg per kg body weight, respectively. Mice received the respective verum compound or the carrier solution (serving as Placebo control) perorally by gavage (in a 0.3 ml volume) once daily starting 2 days before *T. gondii* infection until the end of the experiment at day (d) 8 post infection (p.i.).

### Sampling procedures and determination of small intestinal shortening

Mice were sacrificed by isoflurane 8 days after infection (Abbott, Wiesbaden, Germany). Cardiac blood and tissue samples from liver, spleen, kidneys, mesenteric lymph nodes and terminal ileum were removed under sterile conditions. Intestinal samples from each mouse were collected in parallel for histological, microbiological, immunological and molecular analyses. The relative shortening of the small intestine was calculated by dividing the difference of the mean length of small intestine from age- and sex-matched naïve control mice minus the length of infected mice at day 8 p.i. and then multiplied by 100 over the mean length of naïve control mice small intestines (relative shortening in length  =  (mean d0 – d8 p.i.)×100/mean d0). Results were expressed as % shortage.

### Histologic scores and determination of parasite load

Histologic scores of ileitis and parasite loads were determined in tissue samples from terminal ileum immediately fixed in 5% formalin and embedded in paraffin as described earlier [14], [15]. Sections (5 µm) were stained with hematoxylin and eosin and examined by light microscopy. Our standardized histological inflammation score ranging from 0 to 6 (0, normal; 1, edematous blubbing; 2, cell-free exudate into the lumen, but intact epithelium; 3, cellular shedding into the lumen; 4, beginning epithelial disintegration; 5, mucosal destruction <50% of small intestine length; 6, complete destruction >50% of small intestine length, severe necroses) was used for blinded duplicate evaluation (by M.M.H. and M.M.). Numbers of parasitophorous vacuoles containing tachyzoites or tachyzoite antigens were determined by immunohistology using a rabbit anti-*T. gondii*- IgG antiserum and counted in the terminal ileum, as previously described [21]. In addition, *T. gondii* DNA in ileal biospies was quantified as described earlier [12].

### Immunohistochemistry

In situ immunohistochemical analysis of ileum paraffin sections were performed as described previously [39]. Primary antibodies against CD3 (# N1580, Dako, Glostrup, Denmark, dilution 1∶10), FOXP3 (FJK-16s, eBioscience, San Diego, USA, 1∶100), myeloperoxidase-7 (MPO7, # A0398, Dako, 1∶500), and Ki-67 (TEC3, Dako, 1∶100) were used. For each animal, the average number of positive stained cells within at least six independent high power fields (HPF, high power visual field in 400× magnification) were determined microscopically by two independent investigators (M.M.H. and C.L.) and subjected to statistical analysis as indicated.

### Real-time PCR

RNA was isolated from organs, reverse transcribed and analyzed as described previously [12]. Mouse IL-23p19, IFN-γ and TNF-α mRNA expressions were detected and analyzed using Light Cycler Data Analysis Software, Roche. Expression levels were calculated relative to the HPRT expression (and indicated as “Arbitrary Units”).

### Cytokine detection in ileum, MLN, and spleen culture supernatants

Ileum biopsies were cut longitudinally, washed with PBS and strips of 1 cm^2^, MLNs or spleen placed in 24-flat-bottom well culture plates (Nunc, Wiesbaden, Germany) containing 500 µl serum-free RPMI 1640 medium supplemented with penicillin (100 U/ml) and streptomycin (100 µg/ml; PAA Laboratories). After 18 h at 37°C, culture supernatants were tested for IFN-γ, MCP-1, and IL-10 concentrations by the Mouse Inflammation Cytometric Bead Array (CBA; BD Biosciences).

### Cultural analysis of the ileum microflora and bacterial translocation

Culture-based analysis of the ileum flora was performed as described earlier [14]. For qualitative detection of bacterial translocation, cardiac blood (approx. 0.5 ml) or the respective organ (liver, spleen, MLN, kidney) was immediately transferred into thioglycolate broth under sterile conditions and incubated for maximum 7 days at 37°C. Bacterial growth was monitored daily by turbidity assessment. Aliquots from turbid broths were cultivated on solid media under aerobic, microaerophilic and obligate anaerobic conditions and the bacterial species identified microbiologically and biochemically as described earlier [14].

Antimicrobial effects of Simvastatin, Curcumin and Resveratrol were assessed *in vitro* by inoculating a blank susceptibility disk (Oxoid, Basingstoke, UK) with 10 µl of either compound. Before, the blank disc was placed onto an Müller-Hinton agar plate (Oxoid) that was inoculated with a highly susceptible *Bacillus subtilis* ATCC 6633 strain. After overnight incubation (37°C), lack of growth inhibition around the disk was indicating a negative antimicrobial result [40].

### Statistical analysis

Mean values, medians, standard deviations, standard errors of the mean, and levels of significance were determined using appropriate tests as indicated (Student's t-Test, Mann-Whitney U-Test or the log-rank test for Kaplan-Meier analysis of survival). Two-sided probability (p) values ≤0.05 were considered significant. All experiments were repeated at least twice.

## References

[1] Jain SK (1994). Ethnobotany and research on medicinal plants in India.. Ciba Found Symp.

[2] Kobashigawa JA, Katznelson S, Laks H, Johnson JA, Yeatman L (1995). Effect of pravastatin on outcomes after cardiac transplantation.. N Engl J Med.

[3] Baur JA, Pearson KJ, Price NL, Jamieson HA, Lerin C (2006). Resveratrol improves health and survival of mice on a high-calorie diet.. Nature.

[4] Elmali N, Baysal O, Harma A, Esenkaya I, Mizrak B (2007). Effects of resveratrol in inflammatory arthritis.. Inflammation.

[5] Ma ZH, Ma QY, Wang LC, Sha HC, Wu SL (2005). Effect of resveratrol on peritoneal macrophages in rats with severe acute pancreatitis.. Inflamm Res.

[6] Larrosa M, Yanez-Gascon MJ, Selma MV, Gonzalez-Sarrias A, Toti S (2009). Effect of a low dose of dietary resveratrol on colon microbiota, inflammation and tissue damage in a DSS-induced colitis rat model.. J Agric Food Chem.

[7] Calabrese V, Butterfield DA, Stella AM (2003). Nutritional antioxidants and the heme oxygenase pathway of stress tolerance: novel targets for neuroprotection in Alzheimer's disease.. Ital J Biochem.

[8] Huang TS, Lee SC, Lin JK (1991). Suppression of c-Jun/AP-1 activation by an inhibitor of tumor promotion in mouse fibroblast cells.. Proc Natl Acad Sci U S A.

[9] Sugimoto K, Hanai H, Tozawa K, Aoshi T, Uchijima M (2002). Curcumin prevents and ameliorates trinitrobenzene sulfonic acid-induced colitis in mice.. Gastroenterology.

[10] Simes J, Furberg CD, Braunwald E, Davis BR, Ford I (2002). Effects of pravastatin on mortality in patients with and without coronary heart disease across a broad range of cholesterol levels. The Prospective Pravastatin Pooling project.. Eur Heart J.

[11] Diomede L, Albani D, Sottocorno M, Donati MB, Bianchi M (2001). In vivo anti-inflammatory effect of statins is mediated by nonsterol mevalonate products.. Arterioscler Thromb Vasc Biol.

[12] Munoz M, Heimesaat MM, Danker K, Struck D, Lohmann U (2009). Interleukin (IL)-23 mediates Toxoplasma gondii-induced immunopathology in the gut via matrixmetalloproteinase-2 and IL-22 but independent of IL-17.. J Exp Med.

[13] Liesenfeld O, Kosek J, Remington JS, Suzuki Y (1996). Association of CD4+ T cell-dependent, interferon-gamma-mediated necrosis of the small intestine with genetic susceptibility of mice to peroral infection with Toxoplasma gondii.. J Exp Med.

[14] Heimesaat MM, Bereswill S, Fischer A, Fuchs D, Struck D (2006). Gram-negative bacteria aggravate murine small intestinal Th1-type immunopathology following oral infection with Toxoplasma gondii.. J Immunol.

[15] Heimesaat MM, Fischer A, Jahn HK, Niebergall J, Freudenberg M (2007). Exacerbation of murine ileitis by Toll-like receptor 4 mediated sensing of lipopolysaccharide from commensal Escherichia coli.. Gut.

[16] Maul J, Loddenkemper C, Mundt P, Berg E, Giese T (2005). Peripheral and intestinal regulatory CD4+ CD25(high) T cells in inflammatory bowel disease.. Gastroenterology.

[17] Gerdes J, Li L, Schlueter C, Duchrow M, Wohlenberg C (1991). Immunobiochemical and molecular biologic characterization of the cell proliferation-associated nuclear antigen that is defined by monoclonal antibody Ki-67.. Am J Pathol.

[18] Podolsky DK (2002). Inflammatory bowel disease.. N Engl J Med.

[19] Basset C, Holton J (2002). Inflammatory bowel disease: is the intestine a Trojan horse?. Sci Prog.

[20] Liesenfeld O (2002). Oral infection of C57BL/6 mice with Toxoplasma gondii: a new model of inflammatory bowel disease?. J Infect Dis.

[21] Vossenkamper A, Struck D, Alvarado-Esquivel C, Went T, Takeda K (2004). Both IL-12 and IL-18 contribute to small intestinal Th1-type immunopathology following oral infection with Toxoplasma gondii, but IL-12 is dominant over IL-18 in parasite control.. Eur J Immunol.

[22] Maher BM, Dhonnchu TN, Burke JP, Soo A, Wood AE (2009). Statins alter neutrophil migration by modulating cellular Rho activity–a potential mechanism for statins-mediated pleotropic effects?. J Leukoc Biol.

[23] Jahovic N, Gedik N, Ercan F, Sirvanci S, Yuksel M (2006). Effects of statins on experimental colitis in normocholesterolemic rats.. Scand J Gastroenterol.

[24] Camacho-Barquero L, Villegas I, Sanchez-Calvo JM, Talero E, Sanchez-Fidalgo S (2007). Curcumin, a Curcuma longa constituent, acts on MAPK p38 pathway modulating COX-2 and iNOS expression in chronic experimental colitis.. Int Immunopharmacol.

[25] Ung VY, Foshaug RR, MacFarlane SM, Churchill TA, Doyle JS Oral administration of curcumin emulsified in carboxymethyl cellulose has a potent anti-inflammatory effect in the IL-10 gene-deficient mouse model of IBD.. Dig Dis Sci.

[26] Jobin C, Bradham CA, Russo MP, Juma B, Narula AS (1999). Curcumin blocks cytokine-mediated NF-kappa B activation and proinflammatory gene expression by inhibiting inhibitory factor I-kappa B kinase activity.. J Immunol.

[27] Jian YT, Mai GF, Wang JD, Zhang YL, Luo RC (2005). Preventive and therapeutic effects of NF-kappaB inhibitor curcumin in rats colitis induced by trinitrobenzene sulfonic acid.. World J Gastroenterol.

[28] Martin AR, Villegas I, La Casa C, de la Lastra CA (2004). Resveratrol, a polyphenol found in grapes, suppresses oxidative damage and stimulates apoptosis during early colonic inflammation in rats.. Biochem Pharmacol.

[29] Sanchez-Fidalgo S, Cardeno A, Villegas I, Talero E, de la Lastra CA Dietary supplementation of resveratrol attenuates chronic colonic inflammation in mice.. Eur J Pharmacol.

[30] Fooks LJ, Gibson GR (2002). Probiotics as modulators of the gut flora.. Br J Nutr.

[31] Candela M, Perna F, Carnevali P, Vitali B, Ciati R (2008). Interaction of probiotic Lactobacillus and Bifidobacterium strains with human intestinal epithelial cells: adhesion properties, competition against enteropathogens and modulation of IL-8 production.. Int J Food Microbiol.

[32] Lubbad A, Oriowo MA, Khan I (2009). Curcumin attenuates inflammation through inhibition of TLR-4 receptor in experimental colitis.. Mol Cell Biochem.

[33] Baugh MD, Perry MJ, Hollander AP, Davies DR, Cross SS (1999). Matrix metalloproteinase levels are elevated in inflammatory bowel disease.. Gastroenterology.

[34] Claramunt RM, Bouissane L, Cabildo MP, Cornago MP, Elguero J (2009). Synthesis and biological evaluation of curcuminoid pyrazoles as new therapeutic agents in inflammatory bowel disease: effect on matrix metalloproteinases.. Bioorg Med Chem.

[35] Medina C, Santana A, Paz MC, Diaz-Gonzalez F, Farre E (2006). Matrix metalloproteinase-9 modulates intestinal injury in rats with transmural colitis.. J Leukoc Biol.

[36] Aggarwal BB, Sung B (2009). Pharmacological basis for the role of curcumin in chronic diseases: an age-old spice with modern targets.. Trends Pharmacol Sci.

[37] Rapin JR, Wiernsperger N (2010). Possible links between intestinal permeablity and food processing: A potential therapeutic niche for glutamine.. Clinics (Sao Paulo).

[38] Ahn J, Lee H, Kim S, Ha T (2007). Resveratrol inhibits TNF-alpha-induced changes of adipokines in 3T3-L1 adipocytes.. Biochem Biophys Res Commun.

[39] Heimesaat MM, Fischer A, Siegmund B, Kupz A, Niebergall J (2007). Shift towards pro-inflammatory intestinal bacteria aggravates acute murine colitis via Toll-like receptors 2 and 4.. PLoS One.

[40] Kouri TT, Gant VA, Fogazzi GB, Hofmann W, Hallander HO (2000). Towards European urinalysis guidelines. Introduction of a project under European Confederation of Laboratory Medicine.. Clin Chim Acta.

